# Gender differences in repeat-year experience, clinical clerkship performance, and related examinations in Japanese medical students

**DOI:** 10.1097/MD.0000000000030135

**Published:** 2022-08-19

**Authors:** Nobuyasu Komasawa, Fumio Terasaki, Ryo Kawata, Takashi Nakano

**Affiliations:** a Medical Education Center, Faculty of Medicine, Osaka Medical and Pharmaceutical University.

**Keywords:** clinical clerkship, gender difference, summative tests

## Abstract

While the number of female medical students is increasing in Japan, gender differences in medical school performance have not been studied extensively. This study aimed to compare gender differences in repeat-year experience, Clinical Clerkship (CC) performance, and related examinations in Japanese medical students. We retrospectively analyzed the number of repeat-year students and years to graduation for male and female medical students, and assessed gender differences in performance on computer-based testing (CBT) before CC, CC as evaluated by clinical teachers, the CC integrative test, and the graduation examination in 2018-2020 graduates from our medical school. Subgroup analyses excluding repeat-year students were also performed. From 2018 to 2020, 328 medical students graduated from our medical school. There were significantly fewer repeat-year female students compared to male students (*P* = .010), and the average number of years to graduate was significantly higher for male students than female students (*P* < .001). Female students showed higher scores and performance in all integrative tests and CC (*P* < .05, each). In analysis excluding repeat-year students, there were no significant gender difference in performance on the CBT, and CC integrative test, although female students significantly outperformed male students on the CC and graduation examination. Female medical students had a fewer number of repeat-years and performed better in the CC and graduation examination compared to their male counterparts.

## 1. Introduction

Japan’s national system of modern medical education was developed in the late 1800’s, and was influenced by the German medical education model. In the early 1900’s, there were only a few female doctors. After World War II, the American style of standards-based systematic medical education was introduced, together with several social and cultural concepts.^[1]^ The proportion of female doctors in Japan are continually increasing as well as professions, but still remains the lowest (21–22%) among Organization for Economic Co-operation and Development countries.^[2]^ In Japan, the medical school is 6-year system and candidates who graduated or expected to from high school can take medical school entrance examination which present original their own evaluation standards.

To perform effective curriculum development or learning support to medical students, learning tendency evaluations are important.^[3]^ Some studies have reported associations between demographic factors (e.g., gender and age) and academic performance and dropout and repeat-year rates.^[4,5]^ These studies have focused mainly on academic achievement in the early stages of the medical education curriculum.^[6]^

Clinical medicine, which is the integrative part of medical education curriculum, is a complex academic discipline and one of the major causes of repeat-year which delay their graduation and give much stress.^[7]^ In clinical curriculums, several factors have been reported to impact academic success, including previous academic performance and technical and non-technical skills.^[8,9]^ In USA, there are some studies examined gender difference in clinical clerkship (CC) and USMLE accomplishment.^[10,11]^ In other studies, females reportedly show higher performance in communication skills and information gathering skills,^[12–15]^ while males show higher performance in certain surgical and mathematical skills.^[16,17]^ However, no studies have assessed the performance on CC and related examinations from the viewpoint of gender difference in Japanese medical education context.

Against this backdrop, the present study aimed to compare gender differences in medical students’ performance on the computer-based testing (CBT), CC, integrative test after CC, and graduation exam, as well as the repeat-year incidence of 2018–2020 graduates from our medical school.

## 2. Methods

### 2.1. Ethical consideration and data collection

This study was approved by the Research Ethics Committee of Osaka Medical and Pharmaceutical University (No.2021-002). Oral or written informed consent was unnecessary as this is a retrospective study and set opt-out method. Students were also informed that they had the opportunity to withdraw from the study if they notified the investigator via the university homepage for about 1 month. There were no minors in the study population, since all 4th–6th year medical students in Japan are aged > 20 years. We collected the data from the students’ accomplishment records of our university electronically. Research Ethics Committee of our university permitted to present and analyze the repeat-year number or accomplishments because anonymity is guaranteed. The inclusion criteria was 2018–2020 graduates from our medical school. We did not set exclusion criteria.

### 2.2. Study population

We evaluated 328 students of Osaka Medical and Pharmaceutical University who graduated from 2018 to 2020.

### 2.3. Settings

**Figure 1.**
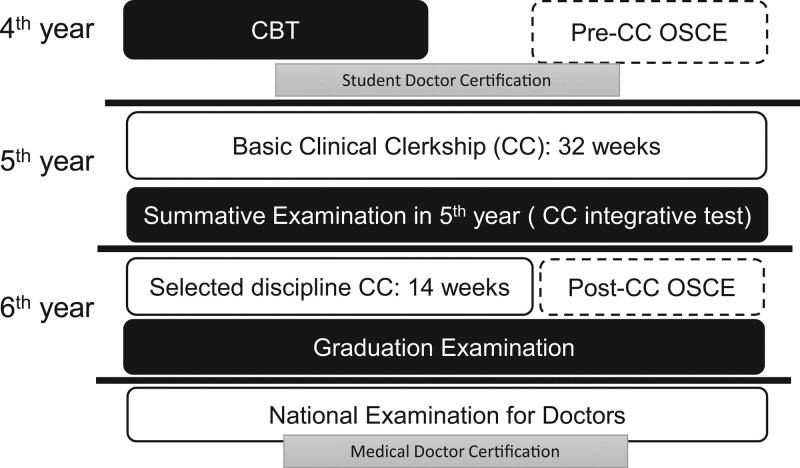
Timeline of medical student curriculum.

As is the case for most medical schools in Japan, Osaka Medical and Pharmaceutical University requires its students to take the CBT in their 4^th^ year, before they enter into CCs in their 5^th^ and 6^th^ years. The 5^th^ year CC integrative test was performed at the end of the 5^th^ year, and the graduation examination was performed in the 6^th^ year (Fig. 1).

### 2.4. CBT content

In 2005, the Common Achievement Test Organization (CATO) was established as a third party and introduced the CBT to evaluate basic medical knowledge before CC in Japan.^[18]^ The CBT consists of multiple-choice 320 questions about basic clinical knowledge over the course of 6 hours. The CBT includes clinical disciplines and related basic medical knowledge.^[19]^

### 2.5. Clinical clerkship (cc) content

Medical students participate in a basic CC during their 5^th^ year. The basic CC involves participation in CCs of all clinical departments of the hospital over the course of 32 weeks. After students complete the basic CC, they then select a discipline they wish to participate for 14 weeks in their 6^th^ year (Fig. 1). CCs recommend medical students to participate as members of a medical team to perform actual medical procedures. The range of medical procedures which can be performed by students is defined and carried out under the supervision of an instructing doctor.^[20,21]^

Supervising doctors of each department evaluate the clinical skills of students utilizing an evaluation sheet based on the Direct Observation of Procedural Skills (DOPS) and mini-CEX.^[22,23]^ Evaluations are based on three essential components: a 5-point evaluation sheet with 16 views (80%), subjective evaluation by the organizer (10%), and a written report (10%) (Fig. 1).

Scores for each CC are collected and we calculated an average score. In our study, we used the basic CC (32 weeks) score, because all medical students are required to participate in the basic CC.

### 2.6. 5th year integrative test

The 5^th^ year integrative test is performed after the basic CC and takes about 7 hours to complete. The test consists of mark sheet-based multiple choice questions and extended matching items, and students are required to answer about 220–230 questions related to clinical knowledge.

### 2.7. 6th year graduation test

The 6^th^ year graduation test involves completing 4 mark sheet-based multiple choice examinations consisting of two 7-hour and two 14-hour integrative exams, for a total of about 1200 questions. The 6^th^ year graduation test consists of multiple-choice questions and extended matching items, and students are required to answer questions about clinical knowledge over the course of 7 hours. The weight of each of the 4 graduation tests is based on a 1:1:4:4 ratio, and the calculated percentage is used in summative evaluation for graduation.

### 2.8. Statistical analysis

Statistical analysis was performed using JMP^®^ 11 (SAS Institute Inc., Cary, NC). Results were compared using the chi-square test or unpaired Student t test. Data are presented as mean ± SD. *P* < .05 was considered statistically significant.

### 2.9. Patient and public involvement

Neither patients nor the public were involved in the design, execution, reporting, or dissemination of this study.

## 3. Results

No graduated asked excluding their data for analysis during the opt-out period. From 2018 to 2020, 328 medical students graduated from our medical school. The number of repeat-year students and average number of years to graduation are shown in Table 1. Female students had significantly fewer numbers of repeat-years and years to graduation compared to male students.

**Table 1:** Number of repeat-year students and years needed to graduate.

Number of graduate students	All n = 328	Male n = 237	Female n = 91	*P*
Repeat-year experience/all	40 (12.2%)	35 (14.8%)	5 (5.5%)	*P* = .010*
Years needed to graduate	6.23 ± 0.80	6.28 ± 0.91	6.09 ± 0.41	*P* < .001*

* *P* < .05 compared by chi-square test or unpaired Student’s *t* test.

Data are presented as mean ± SD or number of students.

Gender comparisons of examination scores and performance evaluations in all students are shown in Table 2. Female students showed superior scores on all integrative tests and performance on CC compared to male ones (Table 2).

**Table 2:** Gender differences in graduation examination, Clinical Clerkship (CC) integrative test, CC performance evaluation, Computer based testing (CBT) percentage, and IRT in all students.

	Graduation Examination (6^th^ year)	CC integrative test (5^th^ year)	CC performance evaluation (5^th^ year)	CBT (4^th^ year)	CBT-IRT
Male N = 237	74.4 ± 5.6	72.4 ± 7.0	78.4 ± 3.0	78.7 ± 8.4	522.7 ± 86.0
Female N = 91	76.4 ± 5.9	74.1 ± 6.7	80.5 ± 2.9	81.1 ± 7.3	546.6 ± 86.5
*P* value	*P* = .002*	*P* = .020*	*P* < .001*	*P* = .010*	*P* = .022*

* *P* < .05 compared by unpaired Student t test.

Data are presented as mean ± SD.

When repeat-year students were excluded, there were no significant differences in CBT% and scores on the IRT and CC integrative test between male and female students, although female students significantly outperformed male students on the CC and graduation examination (Table 3).

**Table 3:** Gender differences in graduation examination, Clinical Clerkship (CC) integrative test, CC performance evaluation, Computer based testing (CBT) percentage, and IRT excluding repeat-year students.

	Graduation Examination (6^th^ year)	CC integrative test (5^th^ year)	CC performance evaluation (5^th^ year)	CBT (4^th^ year)	CBT-IRT
Male N = 202	75.0 ± 5.3	73.2 ± 6.9	78.8 ± 2.8	80.0 ± 7.5	532.1 ± 83.1
Female N = 86	76.6 ± 5.8	74.5 ± 6.5	80.8 ± 2.6	81.4 ± 7.0	547.6 ± 84.2
*P* value	*P* = .022*	*P* = .114	*P* < .001*	*P* = .104	*P* = .116

* *P* < .05 compared by unpaired Student t test.

Data are presented as mean ± SD or number of patients.

## 4. Discussion

In the present study, female students outperformed male students on the graduation examination in both analyses that included and excluded repeat-year students, potentially reflecting their better CC performance. Although performance on the CC integrative test did not significantly differ between male and female students, the better CC performance among female students may have contributed to higher graduation examination scores.

One potential reason for this gender difference is that the graduation examination is constructed based on national examinations for physicians and a deep CC experience cultivates better clinical thinking. Another potential reason to explain the better performance of female students is motivation, which is considered to have a nonnegligible impact on CC performance.^[20,24]^ Future studies assessing the association between CC performance and examination scores are warranted. It will be important for medical teachers to understand these tendencies, identify students at risk for poor academic performance, monitor such students, and provide them with appropriate learning support.

In 2018, events in Japan made world news with shocking headlines. Scores from medical school entrance examinations for female students were manipulated to deliberately exclude them from admission. At least 9 medical schools were involved.^[25]^ One reason of such discrimination was that the medical schools heavily relied on a workforce consisting of their own graduates. Thus, these schools attempted to increase the proportion of male doctors, assuming that female graduates would quit working as full-time employees due to marriage, pregnancy, and childcare. To determine whether similar discrimination might be occurring at other medical schools, the government initiated a nationwide investigation and revealed that many medical schools similarly restricted female student admissions.^[26]^ As medical school entrance examination are performed by its individual manner, the overall gender difference analysis in all medical school entrance exams are difficult to evaluate.

From the viewpoint of career planning, female medical students are motivated to minimize the learning period and maintain a balance between work and life events.^[27]^ In other words, female medical students are prepared to construct seamless under and post graduate career design. One of the main objectives of most medical schools worldwide is to provide students with an education that prepares them to transition seamlessly from the stage of knowledge acquisition to performing practical skills in clinical settings. Thus, it may be important that medical educators to activate more career design program for female medical students.

This study has several limitations worth noting. First, we performed a summative evaluation of CC performance, and other integrative test in a single number though medical students rotate through so many subject areas, are assessed on so many skills.^[28]^ Second, we compared gender differences between clinical portions of the medical education curriculum because no integrative test related to clinical aspects is not performed before then. In the future study, it is warranted to evaluate gender difference in accomplishments in the first 3 years utilizing some scales such as grade point analysis. Finally, as the data came from a single institution, our findings may not be generalizable to other medical schools. However, we believe that our results can be applied to most other Japanese medical schools as they all follow the main core curriculum provided by the Ministry of Education. It will be important to evaluate gender differences in postgraduate clinical performance and their relationships with undergraduate factors in future studies.^[29,30]^

## 5. Conclusion

We conducted a gender difference analysis in 1 Japanese medical schools and found that a fewer number of repeat-year students and better performance on the clinical integrative tests were associated with superior CC performance by female medical students.

All data generated or analyzed during this study are included in this published article [and its supplementary information files].

Supplemental Digital Content is available for this article. The dataset supporting the conclusions of this article is included within the article as additional file (supplementary material, Supplemental Digital Content 1, http://links.lww.com/MD/H53).

## Author contributions

N.K. performed the study, statistical analysis, and wrote the manuscript.

F.T. performed the study and wrote the manuscript.

R.K. performed interpretation of data, prepared the manuscript, and provided critical comments.

T.N. performed interpretation of data, prepared the manuscript, and provided critical comments.

All authors have read and approved the manuscript, and ensure that this is the case.

## Supplementary Material


